# *De novo* HNF1 homeobox B mutation as a cause for chronic, treatment-resistant hypomagnesaemia

**DOI:** 10.1530/EDM-17-0120

**Published:** 2018-03-21

**Authors:** C E Stiles, R Thuraisingham, D Bockenhauer, L Platts, A V Kumar, M Korbonits

**Affiliations:** 1 Department of Endocrinology, William Harvey Research Institute, Barts and the London School of Medicine, Queen Mary University of London, London, UK; 2 Department of Nephrology, Barts Health NHS Trust, London, UK; 3 UCL centre for Nephrology and Great Ormond Street Hospital NHS Trust, London, UK; 4 North East Thames Regional Genetics Laboratory, Great Ormond Street Hospital NHS Trust, London, UK; 5 North East Thames Regional Genetics Service, Great Ormond Street Hospital NHS Trust, London, UK

## Abstract

29-year-old female presenting with an 8-year history of unexplained hypomagnesaemia, which was severe enough to warrant intermittent inpatient admission for intravenous magnesium. Urinary magnesium was inappropriately normal in the context of hypomagnesaemia indicating magnesium wasting. Ultrasound imaging demonstrated unilateral renal cysts and computed tomography of kidneys, ureters and bladder showed a bicornuate uterus. Referral to genetic services and subsequent testing revealed a *de novo HNF1B* deletion.

**Learning points:**

- *HNF1B* loss-of-function mutations are one of the most common monogenic causes of congenital anomalies of the kidney and urinary tract.
- Those with *HNF1B* mutations may have some of a constellation of features (renal and hepatic cysts, deranged liver function tests, maturity onset diabetes of the young type 5 (MODY5), bicornuate uterus, hyperparathyroidism, hyperuricaemic gout, but presenting features are highly heterogeneous amongst patients and no genotype/phenotype correlation exists.
- *HNF1B* mutations are inherited in an autosomal dominant pattern but up to 50% of cases are *de novo.*
- *HNF1B* mutations can be part of the Chr17q12 deletion syndrome, a contiguous gene deletion syndrome.
- Inorganic oral magnesium replacements are generally poorly tolerated with side effects of diarrhoea. Organic magnesium compounds, such as magnesium aspartate, are better absorbed oral replacement therapies.

## Background

This case presentation explores the history and treatment of a patient with an *HNF1B* mutation. This patient presented with hypomagnesaemia in her late teens – a less usual presentation of this condition; cases are more commonly picked up prenatally due to abnormal kidney echogenicity or cysts. We provide a review of the genetic basis for the constellation of features found in association with *HNF1B* mutations, discuss the steps taken to make a diagnosis and provide some guidance on oral magnesium replacement therapies and their relative merits.

## Case presentation

A 29-year-old female presented with an eight-year history of hypomagnesaemia. This had been noted at the age of 21 years whilst being treated for mumps-related pancreatitis. The hypomagnesaemia caused symptoms of headaches and lethargy and replacement with magnesium glycerophosphate 4 mg three times daily had been instituted. It was suspected that her compliance with the medication was poor as the patient still required occasional inpatient admission for symptomatic hypomagnesaemia and received intravenous magnesium infusions.

## Investigation

**Figure 1:**
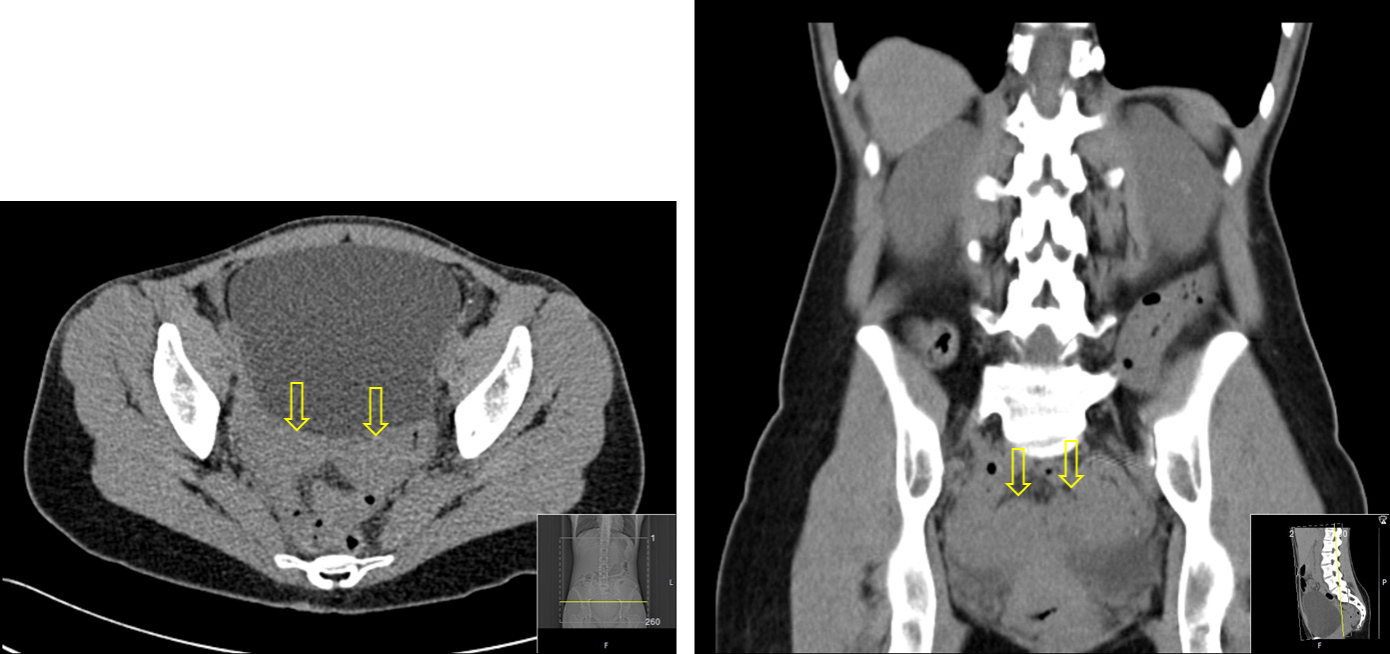
Abdominal CT demonstrating bicornuate uterus. Arrows indicate uterine ‘horns’.

**Figure 2:**
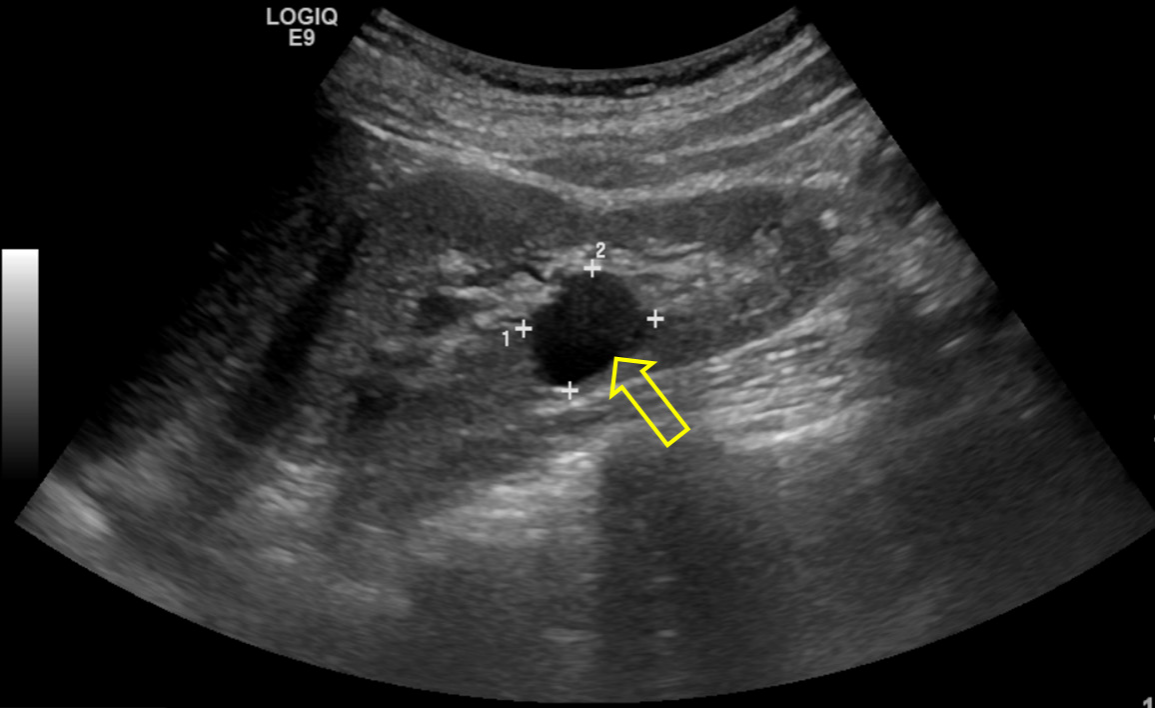
Renal ultrasound showing a renal cyst (arrow).

Serum magnesium was 0.51 mmol/L at presentation to our department, despite the oral replacement therapy. 24-h urinary magnesium (3.7 mmol/day, normal range: 3–5 mmol/day) was noted to be inappropriately normal in the context of low serum magnesium (0.46 mmol/L, normal range: 0.7–1 mmol/L) with hypocalciuria (24-h urinary calcium 0.8 mmol/day, normal range: 2.5–7.5 mmol/day). Serum parathormone was 4.5 pmol/L. Diabetes mellitus was excluded by a normal HbA1c (33 mmol/mol, non-diabetic <42 mmol/mol) and fasting glucose measurement (5 mmol/L, non-diabetic <6.1 mmol/L). Subsequent HbA1c checks have all been within normal range. Estimated glomerular filtration rate was 83 mL/min. CT scanning of the abdomen to exclude renal tract calcification revealed the presence of several hyperdense rounded lesions in the left kidney. The right kidney was normal. A bicornuate uterus was seen (Fig. 1). A subsequent ultrasound (Fig. 2) demonstrated 3 cysts in the left kidney (upper pole 3.1 cm, mid kidney 1.7 cm and lower pole 1.4 cm). The liver, spleen, pancreas and bladder were normal.

## Treatment

Initially, the patient was commenced on oral magnesium glycerol phosphate 4 mg three times daily. However, she remained periodically symptomatic and was admitted periodically for IV magnesium. Serum magnesium ran in the range of 0.46–0.54 mmol/L (normal range 0.7–1 mmol/L). Later, this dose was doubled for a trial period, with no resulting increase in serum magnesium (0.46 mmol/L). The patient was changed to oral magnesium aspartate 10 mmol twice daily with an appreciable increase in serum magnesium levels (0.57–0.61 mmol/L) and reduction of her symptoms.

## Outcome and follow-up

**Figure 3:**
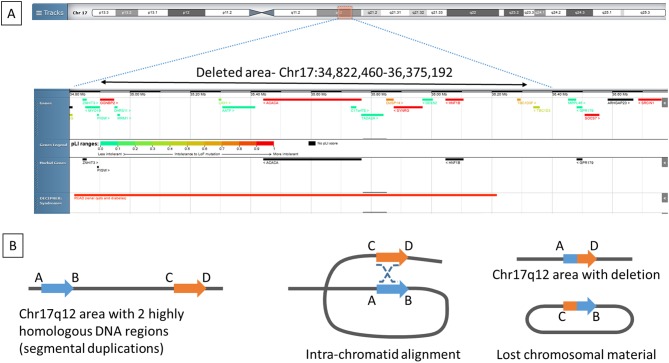
(A) Chromosomal location of the lost chromosomal material on 17q12 (34,822,460-36,375,192; GRCh37/hg19). (B) This region is involved in recurrent deletion mutations as it is flanked on each side by highly repetitive segments of genomic material called segmental duplications (A and B blue arrow and C and D orange arrow). Since these segmental duplications have a high degree of homology to one another, they can misalign during meiosis (middle picture) and give rise to deletions of the intervening genomic interval via non-allelic homologous recombination (marked with staggered lines on the middle picture), resulting in the loss of the same unique genomic region (region B and C on the right picture) in different individuals causing the 17q12 recurrent deletion syndrome (26). Figure was drawn based on illustration in Chen *et al*. (27).

Follow-up has been over a period of 9 years. Referral was made to a genetic testing service, where the patient was tested for an *HNF1B* mutation. A heterozygous whole gene deletion was identified in *HNF1B*. Subsequent array-based comparative genomic hybridization analysis demonstrated a 1.5 Mb deletion within chromosome 17q12 (34,822,460-36,375,192, GRCh37/hg19) (Fig. 3). Neither parent shared the deletion indicating that it was a *de novo* event in our patient. More recently, the patient has sought advice on conception and has been referred for pre-implantation screening to eliminate the risk of transmission of the *HNF1B* mutation.

## Discussion

This patient had a heterozygote large deletion on chromosome 17q12 encompassing several genes (*AATF, ACACA, C17ORF78, DDX52, DHRS11, DUSP14, GGNBP2, HNF1B, LHX1, MRM1, MYO19, PIGW, SYNRG, TADA2A* and *ZNHIT3*). One of the genes in this region *HNF1B*, coding for hepatocyte nuclear factor 1 homeobox beta, is made up of nine coding exons. *HNF1B* mutations (alongside paired box 2 gene *PBX2*) are the most common monogenic cause of congenital anomalies of the kidney and urinary tract (CAKUT) (1), and it is found in 15–23% of CAKUT patients (2, 3, 4). Targeted knockdown of *Hnf1b* in mice results in liver cysts (5) and renal cysts (6). *HNF1B* is known to regulate transcription of polycystic kidney and hepatic disease (*PKHD1*), uromodulin (*UMOD*) and polycystic kidney disease 2 (*PKD2*) (7). Mutations of these genes are known to result in renal cystic diseases (7). *LHX1*, coding for LIM homeobox 1, is also found in the 17q12 area and heterozygote variants are known to cause Müllerian duct abnormalities/Mayer-Rokitansky-Küster-Hauser syndrome (8).

A typical facial phenotype is observed in some patients with 17q12 deletions – high rounded forehead, arched eyebrows, small chin/set back lower jaw and downward slanting eyes. In retrospect, our patient has the latter two characteristics. Less commonly, they may be of short stature with height in the lower 3% of population and have spinal curvature (9). Our patient was shorter than average (152 cm) but was not in the bottom 3rd centile for height and she had exceeded mid-parental height prediction (father 159.5 cm, mother 150 cm, predicted height 148 cm).

### Hypomagnesaemia

The occurrence of hypomagnesaemia is described with various types of *HNF1B* mutations (7, 10, 11). It is believed to occur through magnesium wasting in the renal distal convoluted tubule. The co-existence of hypermagnesuria and hypo/normocalciuria helps to localise its site of the dysfunction (7). Magnesium is predominately reabsorbed in the thick ascending limb of the loop of Henle and the distal convoluted tubule. Magnesium and calcium reabsorption in the thick ascending limb both rely on the adequate function of the intercellular tight junction proteins claudin 16 and 19. Failure of these prevents paracellular passage of both calcium and magnesium and results in the syndrome of familial hypercalciuric hypomagnesemia with nephrocalcinosis (12). As hypercalciuria is not accompanying hypomagnesaemia in *HNF1B* mutation patients, the site of abnormal magnesium handling is unlikely to be in the thick ascending limb .

**Figure 4:**
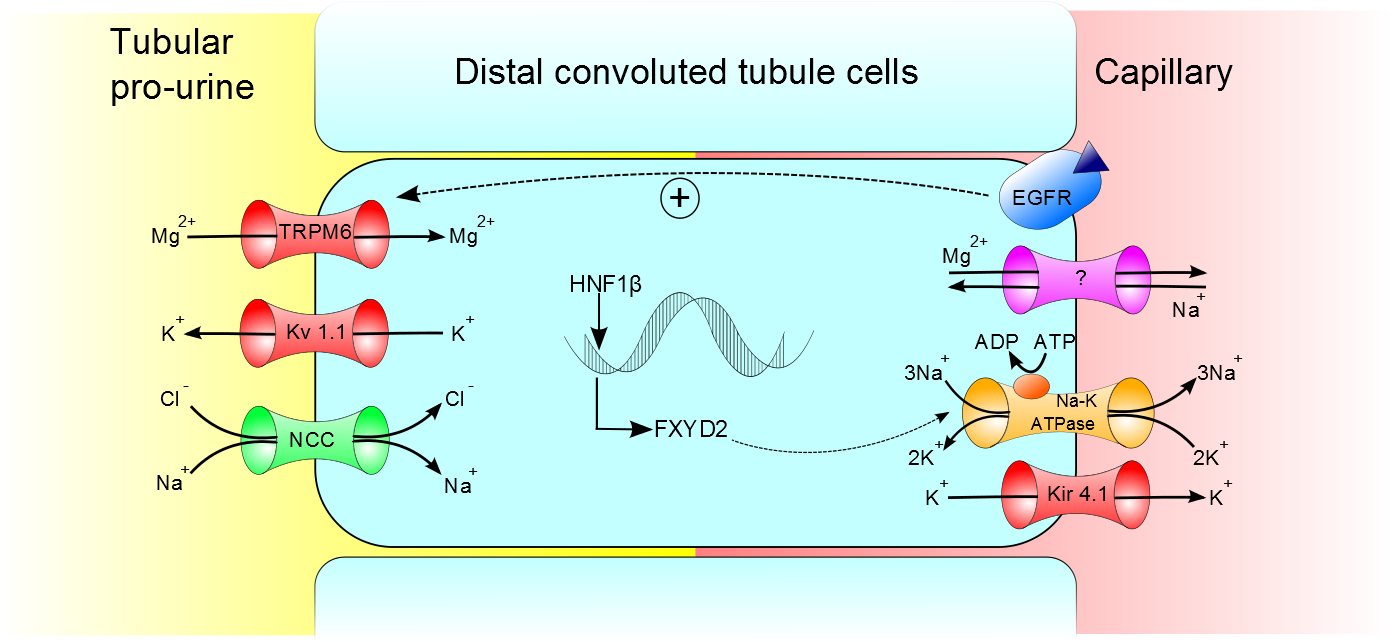
Mechanism of magnesium reabsorption into DCT cells and proposed mechanism for extrusion into the capillaries. Green channel, co-transporter; red channel, ion channel; orange channel, sodium potassium ATPase pump and purple channel, proposed mechanism for magnesium extrusion. EGFR, epidermal growth factor receptor.

In the distal convoluted tubule, *HNF1B* binds to the promoter of the FXYD domain containing ion transport regulator 2 (*FXYD2*) gene (7), which encodes the γ-subunit of a basal membrane Na^+^/K^+^-ATPase in the kidney. This γ-subunit helps to stabilise the α-subunit within the Na^+^/K^+^-ATPase. Loss of the γ-subunits affect the Na^+^-, K^+^- and ATP-binding affinities of the Na^+^/K^+^-ATPase (13). Although the exact method of extrusion of magnesium from the cells of the distal convoluted tubule into the circulation is not yet known, one possibility is that reduced activity in this Na^+^/K^+^-ATPase pump and a consequent reduction in Na^+^ removal from the cell, results in a higher intracellular Na^+^ concentration. This could then prevent its exchange for intracellular Mg^2+^ through the soluble carrier family 41 member A1 (SLC41A1) (14) or A3 (SLC41A3) (15, 16). Mechanisms believed to be involved in the re-absorption of magnesium in the distal convoluted tubule and its extrusion into the capillaries are shown in Fig. 4. We believe, our patient loses magnesium via the distal convoluted tubule.

### Oral magnesium replacement

Our patient was not well controlled on magnesium glycerophosphate while she had significantly better control on magnesium aspartate. In a study using rats, magnesium was better absorbed from organic magnesium salt replacements (magnesium aspartate and particularly magnesium gluconate) than from inorganic salt replacements (e.g. magnesium sulphate and magnesium carbonate) (17). That some magnesium salts are better absorbed than others is reflected in our patient in respect of higher serum magnesium levels upon switching replacement regime from magnesium glycerophosphate to magnesium aspartate. The estimated average requirement for magnesium for a 19- to 30-year-old female is 255 mg (10.6 mmol)/day. This increases to 290 mg (12.7 mmol)/day in pregnancy and 255 mg (11.3 mmol/day) in lactation (18). Our patient may therefore benefit from a small amount of additional magnesium therapy during these periods if she achieves conception.

### MODY5

Although not seen in our patient, *HNF1B* mutations can also cause maturity onset diabetes of the young type 5 (MODY5). This is again thought to be mediated by the formation of cysts – this time in the pancreas, where mouse models have shown a reduction in expression of transcription factors downstream of *Hnf1b*, which are involved in the formation of pancreatic ducts and the development of endocrine cells. A reduction in the number of multipotent progenitor cells has also been noted, which resulted in pancreatic hypoplasia (19). In one study, it was noted that MODY5 generally occurred before the age of 30 years (20); therefore, our patient’s chances of developing diabetes mellitus are now probably lower. We will follow her glycaemic state with annual HbA1c and fasting glucose levels.

### Uterine anomalies and reproductive considerations

*LHX1*, also located in the deleted area, is a transcription factor, which is essential for the normal development and elongation of the female reproductive tract (21). Interestingly, HNF1B** can activate LHX1 (22). Patients with *HNF1B *point mutations, therefore without 17q12 deletion and normal *LHX1*, can show genital abnormalities (23). In our patient, both mechanisms – loss of *LHX1* or loss of HNF1B-induced stimulation of the *LHX1* promoter – could play a role in the development of the uterine abnormality. Bicornuate uterus is one of the most common uterine malformations (24), and it is associated with a spontaneous abortion rate of 36%, a pre-term birth-rate of 23% and a live birth-rate of 55.2% (24, 25).

In summary, the heterozygous large deletion of 17q12 area explains the complex phenotype of this patient.

## Declaration of interest

The authors declare that there is no conflict of interest that could be perceived as prejudicing the impartiality of the research reported.

## Funding

This work was supported by a Wellcome Trust Clinical Training fellowship to CES (grant number 097970/Z/11/Z).

## Acknowledgements

We are grateful for Professor William Fraser (University of East Anglia, Norwich, UK) for the helpful discussions regarding this patient.

## Patient consent

Written, informed consent has been obtained from the patient.

## Author contribution statement

Stiles C E: Clinical lecturer in endocrinology, wrote the text for this case presentation; Thuraisingham R: renal physician consulted on mechanisms for renal magnesium wasting; Bockenhauer D: expert in the area of the *HNF1B* mutations and renal disease, aided in diagnosis of patient; Platts L: responsible for carrying out array CGH confirming 17q12 deletion; Kumar A V:geneticist who performed genetic testing and counselled patient; Korbonits M: supervising consultant, primary physician involved in the patient’s care and in drafting this text.
